# Valorization of Nam Wah Banana (*Musa paradisiaca* L.) Byproducts as a Source of Bioactive Compounds with Antioxidant and Anti-inflammatory Properties: In Vitro and In Silico Studies

**DOI:** 10.3390/foods12213955

**Published:** 2023-10-29

**Authors:** Ansella Amanda Epifani Widoyanti, Kamonwan Chaikong, Panthakarn Rangsinth, Patcharaporn Saengratwatchara, George Pak-Heng Leung, Anchalee Prasansuklab

**Affiliations:** 1 Graduate Program in Public Health Sciences, College of Public Health Sciences, Chulalongkorn University, Bangkok 10330, Thailand; ansellaamanda@gmail.com; 2 Graduate Program in Clinical Biochemistry and Molecular Medicine, Department of Clinical Chemistry, Faculty of Allied Health Sciences, Chulalongkorn University, Bangkok 10330, Thailand; kamonwan.chaikhong@gmail.com (K.C.); p.saengratwatchara@gmail.com (P.S.); 3 Department of Pharmacology and Pharmacy, The University of Hong Kong, Hong Kong SAR, China; ptkrs@hku.hk (P.R.); gphleung@hku.hk (G.P.-H.L.); 4 Faculty of Pharmacy, Payap University, Chiangmai 50000, Thailand; 5 College of Public Health Sciences, Chulalongkorn University, Bangkok 10330, Thailand; 6 Natural Products for Neuroprotection and Anti-ageing (Neur-Age Natura) Research Unit, Chulalongkorn University, Bangkok 10330, Thailand

**Keywords:** Nam Wah banana, agricultural waste, value addition, medicinal properties, lipoxygenase inhibitor, toxicity

## Abstract

Nam Wah banana (*Musa paradisiaca* L.) is the most common banana cultivar in Thailand. Large amounts of its non-consumable byproducts are considered undervalued and thrown as waste. Exploring the potential utilization and application of banana byproducts for human benefit can add to their value and minimize the risk of threats. This study aimed to investigate phytochemicals, antioxidant and anti-inflammatory activities, and toxicity of Nam Wah banana byproducts. Five banana plant parts, including the midrib, leaf, peduncle, unripe and ripe peels, were extracted using hexane, ethyl acetate, ethanol, and water. Among the extracts tested, the ethyl acetate leaf extract showed the strongest antioxidant capacity and anti-inflammatory activity, probably through the inhibition of inducible nitric oxide synthase (iNOS) and 15-lipoxygenase (15-LOX). Positive correlations existed between the activities and the total phenolic/flavonoid content of banana byproducts. An in silico docking analysis demonstrated that flavonoid glycosides in banana byproducts, such as kaempferol-3-O-rutinoside and rutin, may bind to inducible iNOS, whereas omega-3-polyunsaturated fatty acids, such as eicosapentaenoic acid, may bind to 15-LOX and cyclooxygenase-2 (COX-2). The extracts showed either low or no toxicity. These findings suggest that banana byproducts are a natural source of antioxidant and anti-inflammatory compounds. It is recommended that additional investigations be conducted to explore their potential therapeutic applications in treating disorders linked with oxidative stress or inflammation. This research has the potential to enhance the value of banana byproducts.

## 1. Introduction

Bananas are primarily produced in Asia, South America, and Africa. According to the statistical data reported by the Food and Agriculture Organization of the United Nations (FAO), the worldwide production of bananas has increased from 2017 to 2021 at a 5-year average annual rate of 2.2%, reaching 125 million tons in 2021, with last year’s increasing rate of 10.4% [1]. Nam Wah banana (*Musa paradisiaca* L.) is a banana variety that is widely consumed in Thailand and Southeast Asian regions [2]. Bananas have numerous pharmacological activities such as antimicrobial, anticancer, antidiabetic, and antiulcer effects [3]. Bananas are considered household fruits and are commonly consumed fresh. However, some industries process and transform banana fruits into other products, such as banana syrup, frozen bananas, jellies, and banana vinegar [4]. 

Alongside the mass production of banana products, enormous amounts of banana byproducts are inevitably generated. Typically, after fruit harvesting, other non-consumable banana components, such as peels, stems, leaves or any other parts of the banana plant that are not useful in manufacturing processes, are regarded as byproducts and discarded as waste [4,5]. Based on a recent report, an enormous quantity of banana byproducts, estimated to be around 114 million tons on a global scale, was discarded [4]. Agricultural waste is generally considered an undervalued item with low economic potential [6]. The growing production of agricultural waste from banana byproducts can lead to long-term environmental concerns by increasing the production of greenhouse gases in the atmosphere. Continuous exposure to these harmful chemicals can exacerbate human health problems, particularly the development of chronic respiratory diseases, allergies, cardiovascular conditions, and reproductive problems [7]. The incineration of crop residues may create hazardous substances, such as nitrous oxide and carbon monoxide [4]. The aerobic decomposition of fruit waste, such as banana peels, can produce volatile organic compounds that require more awareness in terms of health [8].

Phytochemicals are important secondary metabolites naturally produced by plants for their defense mechanisms. A large number of phytochemical compounds have been found to exhibit numerous biological and pharmacological properties that greatly benefit human health [9,10,11,12,13]. The plant-based byproducts and waste are also rich sources of biologically active compounds with promising therapeutic potential for human diseases [14,15]. In 2019, it was reported by the Department of Agricultural Extension, Ministry of Agriculture and Cooperatives, that the total production of Nam Wah banana in Thailand was around 66 thousand tons [16]. Nakhon Sawan province is regarded as the main agricultural production area in Thailand, with approximately half the population engaged in agricultural work. Notably, the statistical data in 2016 and 2019 showed that the harvested area of Nam Wah banana crops in this province was among the top ten provinces in the country [16,17,18]. Based on these facts, the large amounts of Nam Wah banana waste and byproducts produced in this province may pose a threat to the environment and human health. Thus, exploring the potential utilization and application of this banana’s byproducts for human benefit can help add to their commercial value and minimize the risk of threats. However, the research on this aspect of the byproducts derived from Nam Wah banana still needs to be completed. Moreover, most of the studies on banana species focused on two major parts, the leaf and peel [19], while the midrib and peduncle have yet to attract much attention from researchers. Some biological activities reported in banana (*Musa* spp.) waste include antioxidant, antidiabetes, anticancer, antibacterial, and cardioprotection [19]. Therefore, this study aimed to evaluate the potential value of Nam Wah banana byproducts collected from Nakhon Sawan province, Thailand. Five parts of the banana plant (midrib, leaf, peduncle, unripe peel, and ripe peel) that are commonly discarded in the industry were selected. The phytochemical contents, in vitro antioxidant and anti-inflammatory effects, and toxicity of the extracts were investigated and compared. Correlation analysis and in silico molecular docking were performed to identify the possible active ingredients in banana byproduct extracts. To our knowledge, this is among the few studies to investigate and provide insights into the phytochemical analysis, pharmacological properties, and safety of Nam Wah banana byproducts.

## 2. Materials and Methods

### 2.1. Chemicals and Reagents

Gallic acid, quercetin, diclofenac sodium salt, 2,2-diphenyl-1-picrylhydrazyl (DPPH), L-ascorbic acid, 2,4,6-tripyridyl-s-triazine (TPTZ), sodium nitroprusside (SNP), naphthyl-ethylene diamine dihydrochloride (NED), sulfanilamide, soybean 15-lipoxygenase (LOX), and linoleic acid were purchased from Sigma-Aldrich (St. Louis, MO, USA). The Folin–Ciocalteu reagent was purchased from Merck (Darmstadt, Germany). Aluminum chloride was purchased from Ajax Finechem (Sydney, NSW, Australia). Dimethyl sulfoxide (DMSO), hexane, ethanol, and ethyl acetate were purchased from RCI LabScan (Bangkok, Thailand).

### 2.2. Collection of Plant Materials

Five banana (*M. paradisiaca* L.) byproducts, including the midrib, leaf, unripe peel, ripe peel, and peduncle, were collected from Nakhon Sawan province in Thailand. The banana plant was identified at the Professor Kasin Suvatabandhu Herbarium (Department of Botany, Faculty of Science, Chulalongkorn University) as Musa (ABB) ‘Namwa Sai Lueang,’ and the specimen was deposited with the voucher number 17138 (BCU).

### 2.3. Preparation of Banana Plant Extracts

The banana byproducts were cleaned, oven-dried at 60 °C until a constant weight was obtained, and ground into a fine powder for further extraction. Five grams of the dried powder was sequentially extracted with 300 mL of hexane, ethyl acetate, and ethanol in a Soxhlet apparatus and allowed to proceed for 18 h to 3 days until each extraction solvent was clear. The solvent in the crude extracts was removed using a rotary evaporator. The second extraction was performed using maceration with water. The dried powder (5 grams) was soaked in water (250 mL) and continuously shaken for 24 h. The resulting extract was filtered, and the water was removed by freeze-drying. 

### 2.4. Qualitative Phytochemical Analysis

The presence of phenolic compounds, flavonoids, tannins, diterpenes, steroids, triterpenes, saponins, alkaloids, anthraquinones, and cardiac glycosides in banana byproducts was analyzed using standard qualitative tests [20]. The original protocol was slightly modified by dissolving all extract samples in DMSO before assays and changing the solvent from benzene to chloroform in the anthraquinone test.

### 2.5. Quantitative Phytochemical Analysis

#### 2.5.1. Total Phenolic Content

Total phenolic content was determined using the Folin–Ciocalteu reagent method in a 96-well microplate format [21]. The extract or standard (gallic acid) was pipetted into a microplate well containing 10% Folin–Ciocalteu reagent. After 20 min of incubation, sodium carbonate solution was added, and the mixture was incubated at room temperature for an additional 20 min. The absorbance was measured at 760 nm using a microplate reader (BioTek, Winooski, VT, USA). The total phenolic content was calculated based on the calibration curve of gallic acid and expressed as milligrams of gallic equivalent (GAE) per gram of extract. 

#### 2.5.2. Total Flavonoid Content

Total flavonoid content was determined using an aluminum chloride colorimetric test [21]. Total flavonoid content was calculated using quercetin as the standard and expressed as quercetin equivalent (QE) in mg/g extract. In this assay, the extract or standard was added to a well containing 10% aluminum chloride, absolute ethanol, and 1 M sodium acetate. The mixture was incubated for 40 min at room temperature in the dark. Absorbance was measured at 415 nm using a microplate reader (BioTek). 

### 2.6. Determination of Antioxidant Activity

#### 2.6.1. DPPH Scavenging Activity Assay

The free radical scavenging ability of the extract was assessed using the DPPH assay as previously described with a slight modification [21]. Initially, the extract was added to a 96-well microplate containing DPPH radicals in methanol. The mixture was incubated at room temperature in the dark for 30 min. After incubation, the absorbance was measured at 517 nm using a microplate reader (BioTek). Ascorbic acid was used as the standard, and methanol with DPPH was used as the control. The radical scavenging activity was calculated using Equation (1). The results were expressed as IC_50_ values and vitamin C equivalent antioxidant capacity (VCEAC) per gram of dry extract.

(1) $$Radical\;scavenging\;activity\;\left(\%\right)=\frac{Abs\;Control-Abs\;Sample}{Abs\;Control}\times 100$$

#### 2.6.2. Ferric Reducing Antioxidant Power (FRAP) Assay

The presence of antioxidants was determined by the reduction of the ferric 2,4,6-tripyridyl-s-triazine complex (Fe^3+^-TPTZ) to its ferrous form (Fe^2+^-TPTZ). The FRAP assay was performed as previously described [22], with some modifications. The FRAP solution was prepared by mixing 300 mM acetate buffer (pH 3.6), 10 mM TPTZ, and 20 mM FeCl_3_.6H_2_O at a ratio of 10:1:1. Ascorbic acid was used to establish the standard curves. The reaction was initiated by adding the FRAP reagent to the extract or standard solution. The absorbance was measured at 595 nm after 4 min of incubation using a microplate reader (BioTek). The FRAP value was calculated and expressed as mM ascorbic equivalents (AAE) per milligram of dry extract.

### 2.7. Determination of Anti-Inflammatory Activity

#### 2.7.1. Nitric Oxide (NO) Radical Scavenging Assay

The NO radical scavenging assay was performed using the Griess–Illosvory reaction [23] with some modifications. NO radicals were generated by mixing SNP solution in phosphate-buffered saline (PBS; pH 7.4) with the extract or positive control (gallic acid), followed by incubation under light for 90 min. The mixture was then added and incubated with 1% sulfanilamide for 10 min and subsequently with 0.1% NED for an additional 30 min. Finally, absorbance was measured at 550 nm using a microplate reader (BioTek). The scavenging activity was calculated using Equation (1).

#### 2.7.2. 15-LOX Inhibition Assay

This assay was performed according to the procedure described in a previous study [24] with slight modifications. Initially, 2500 U/mL of 15-LOX in 0.2 M borate buffer (pH 9.0) was mixed with the extract or standard inhibitor (that is, sodium diclofenac) prior to incubation for 15 min at room temperature. The linoleic acid solution was added to the mixture and incubated for an additional 10 min. Finally, the absorbance was measured at 234 nm using a microplate reader (BioTek). 15-LOX inhibitory activity was calculated using Equation (2).

(2) $$15-LOX\;inhibitory\;activity\;\left(\%\right)=\frac{Abs\;control-Abs\;sample}{Abs\;control}\times 100$$

### 2.8. Molecular Docking

Phytochemicals previously reported in the leaves of *Musa* spp. [25,26,27,28] (Supplementary Table S1) were selected and analyzed against target enzymes involved in inflammation (that is, inducible nitric oxide synthase [iNOS], 15-LOX, and cyclooxygenase-2 [COX-2). Compound structures were generated from SMILES strings using BIOVIA Draw 2019 (BIOVIA, San Diego, CA, USA). The X-ray crystal structures of iNOS (PDB ID:3E6T) [29], 15-LOX (PDB ID:1LOX) [30], and COX-2 (PDB ID:5KIR) [31] were purchased from the RCSB Protein Data Bank. The preparation of the ligand and protein structures and the docking process were modified from our previous publication [21]. Briefly, the docking study was performed using the Lamarckian Genetic Algorithm with default parameters in AutoDockTools-1.5.6 software (The Scripps Research Institute, San Diego, CA, USA). Grid sites were set with a spacing of 0.375 Å. The x–y–z dimensions for iNOS, 15-LOX, and COX-2 were set to 40 × 40 × 40 points. Grid box of the x, y, and z centers were 122.657, 114.042, and 36.638 for iNOS, −27.566, 150.834, and 56.884 for 15-LOX, and 23.287, 0.439, and 34.435 for COX-2. Protein–ligand interactions were further visualized and analyzed using the Discovery Studio Visualizer.

### 2.9. Determination of Toxicity Using Brine Shrimp Lethality Test

The method was performed according to the procedure described [32,33] with slight modifications. Artificial seawater was oxygenated by strong aeration and circulation for 24 h before hatching the brine shrimp eggs. After 48 h of hatching, the brine shrimps were transferred to vials containing artificial seawater. Extracts of banana byproducts at various concentrations (10, 100, 250, 500, and 1000 µg/mL) were added. The vials were then placed under a lamp. The DMSO-treated group served as the negative control. After treatment of 24 h, the number of survivors in each group was counted, and the death rate was calculated using Equation (3).

(3) $$Death\;\left(\%\right)=\frac{total-survivor}{total}\times 100$$

### 2.10. Statistical Analysis

All experiments were performed at least thrice, and the results were expressed as the mean ± standard deviation (SD). GraphPad Prism version 9.0 software was used to analyze the raw data and calculate the value of IC_50_. One-way analysis of variance (ANOVA) followed by Tukey’s post hoc test was performed to determine the mean differences among the groups. Pearson’s correlation coefficients (*r*) were used to determine the correlations between distinct variables. Statistical significance was set at *p* < 0.05.

## 3. Results

### 3.1. Extraction Yields of Banana Byproducts

The percentage yields differ among the different extracts. The highest extraction yield is obtained from peduncle banana extraction with water maceration (24.34%), followed by ripe peel water and ethanol extracts (23.46% and 22.35%, respectively), whereas unripe banana peel in the ethyl acetate extract exhibits the lowest yield (1.27%) (Supplementary Table S2).

### 3.2. Phytochemical Analysis of Banana Byproducts

Qualitative chemical analysis was performed to identify the presence of phenolic compounds, flavonoids, tannins, diterpenes, steroids, triterpenes, saponins, alkaloids, anthraquinones, and cardiac glycosides in banana byproducts. The results are summarized in Table 1. The ethyl acetate extract of banana byproducts contains a greater variety of phytochemicals than the other extracts. None of the extracts contain any anthraquinones.

### 3.3. Total Phenolic and Flavonoid Contents

The total phenolic and flavonoid contents of the banana byproducts are shown in Table 2. The ethyl acetate extract of the banana leaf has the highest total phenolic content (148.21 ± 12.04 mg GAE/g dry extract), followed by the ethanolic extract of the unripe peel banana (59.14 ± 2.14 mg GAE/g dry extract). The lowest phenolic content is detected in the water extract of the unripe peel (2.71 ± 0.22 mg GAE/g dry extract).

The ethyl acetate extract of the banana leaf contains the highest total flavonoid content (53.87 ± 5.38 mg QE/g dry extract), followed by the water extract of the banana leaf (22.16 ± 1.65 mg QE/g dry extract). The lowest level of flavonoid content is detected in the water extract of the peduncle banana (1.03 ± 0.19 mg QE/g dry extract). The total flavonoid content of the hexane extract of the unripe banana peel is undetectable.

### 3.4. Antioxidant Activity

#### 3.4.1. DPPH Scavenging Assay

The DPPH radical scavenging activities of the banana byproducts are summarized in Table 3. The ethyl acetate extract of the banana leaf demonstrates the highest DPPH radical scavenging activity with IC_50_ of 0.251 mg/mL and the VCEAC of 235.10 ± 12.88 mg/g dry extract, followed by the ethanol extract of the leaf, the ethanol extract of the unripe peel, and the water extract of the leaf with an IC_50_ of 0.852, 0.968, and 0.978 mg/mL, respectively. The lowest DPPH radical scavenging activity is observed in the water extract of the unripe peels, which exhibits a scavenging activity of <1% at 5 mg/mL. 

#### 3.4.2. Ferric Reducing Antioxidant Power Assay

The antioxidant activities of banana byproducts were also assessed using the FRAP assay. The results are summarized in Table 3. The ethyl acetate extract of the banana leaf exhibits the highest reducing power activity (70.74 ± 0.67 mM AAE/mg dry extract), followed by the ethanolic extract of the banana leaf and the ethanolic extract of the unripe peel (40.62 ± 1.11 and 25.73 ± 1.21 mM AAE/mg dry extract, respectively). The lowest reducing power activity was measured in the water extract of the unripe peel (1.26 ± 0.24 mM AAE/mg dry extract).

### 3.5. Anti-Inflammatory Activity

#### 3.5.1. NO Scavenging Activity Assay

The NO scavenging activities of the banana byproducts are presented in Table 4. At the concentration of 1 mg/mL, the NO scavenging activities of midrib (hexane, ethanol, and water extracts), peduncle (hexane and water extract), unripe peel (hexane and water extract), and ripe peel (hexane and water extract) are not detectable, whereas the NO scavenging activity of the other extracts range from 2.93% ± 1.04–58.34% ± 2.23. The water extract of unripe peel and hexane extract of ripe peel show negligible NO scavenging activity, even at a concentration of 5 mg/mL. Gallic acid (GA) was used as the positive control, which showed an IC_50_ of 0.057 ± 0.003 mg/mL. The highest scavenging activity was determined in the ethyl acetate extract of the banana leaf with an IC_50_ of 0.782 ± 0.03 mg/mL, followed by the hexane extract of the leaf with an IC_50_ 2.744 ± 0.10 mg/mL.

#### 3.5.2. 15-LOX Inhibition Assay

The 15-LOX inhibitory activities of the banana byproducts are presented in Table 5. The ethyl acetate extract of the banana leaves inhibits 15-LOX by 53%, which is the strongest among all the extracts tested. This inhibitory effect is greater than that of the positive control, sodium diclofenac, at the same concentration (which inhibits 15-LOX by 42%). The second most active extract is the hexane extract of the banana leaves, which inhibits 15-LOX activity by 50%. The aqueous extract of the unripe peel shows the lowest activity, with only 10% inhibition.

### 3.6. Correlation Analysis

**Figure 1:**
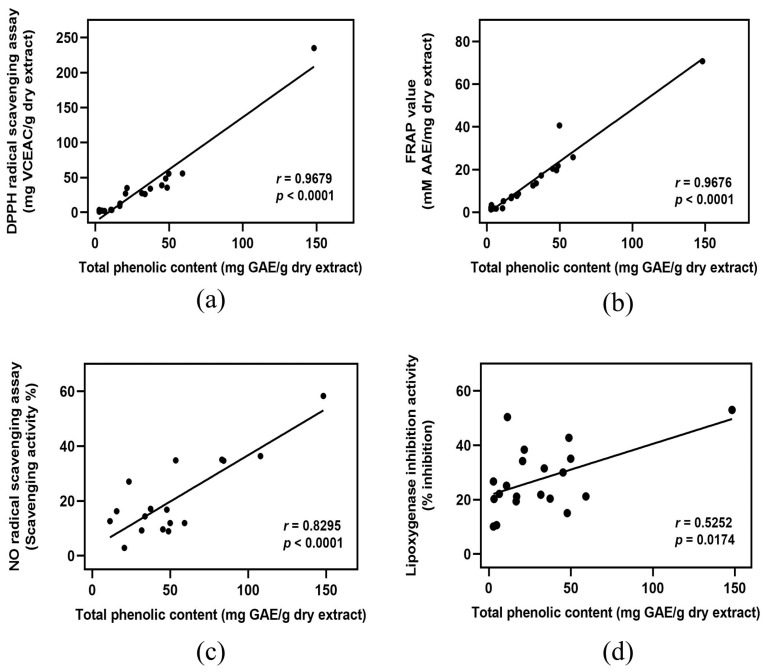
Correlation analysis between total phenolic contents of banana byproducts and their antioxidant and anti-inflammatory activities. Total phenolic content versus (**a**) DPPH radical scavenging activity, (**b**) FRAP value, (**c**) NO radical scavenging activity, and (**d**) 15-LOX inhibition activity. *r*: Pearson’s correlation coefficient.

**Figure 2:**
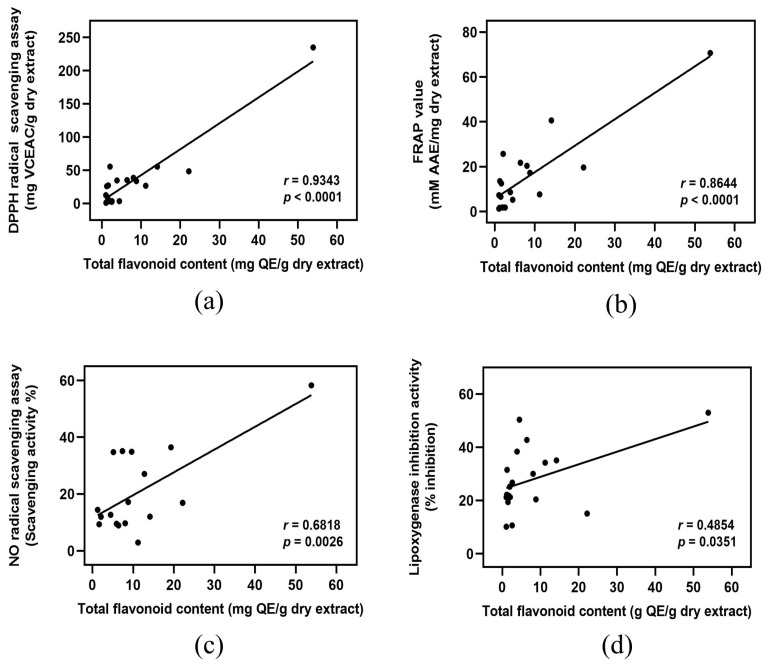
Correlation analysis between total flavonoid contents of banana byproducts and their antioxidant and anti-inflammatory activities. Total flavonoid content versus (**a**) DPPH radical scavenging activity, (**b**) FRAP value, (**c**) NO radical scavenging activity, and (**d**) 15-LOX inhibition activity. *r*: Pearson’s correlation coefficient.

Considering the reported antioxidant and anti-inflammatory capacities of phenolic and flavonoid compounds [34], it was hypothesized that the extract of banana byproducts with higher phenolic and flavonoid contents is likely to have better antioxidant and anti-inflammatory activities. Pearson’s correlation analysis was performed to test this hypothesis. The strength of the association could be reflected in the values of the correlation coefficient (*r* as follows: *r* = 0.000 to 0.200, indicating a negligible correlation; *r* = 0.210 to 0.400, indicating a weak correlation; *r* = 0.410 to 0.700, indicating a moderate correlation; *r* = 0.710 to 0.900, indicating a high correlation; and *r* = 0.910 to 1.000, indicating a strong correlation [35]. The total phenolic content is strongly correlated with DPPH scavenging activity and FRAP value (Figure 1a,b), whereas it shows a high and moderate correlation with NO scavenging activity and 15-LOX inhibition, respectively (Figure 1c,d). Similarly, the total flavonoid content has a strong correlation with DPPH scavenging activity (Figure 2a) and a high correlation with the FRAP value (Figure 2b). However, the total flavonoid content is moderately correlated with NO scavenging activity and 15-LOX inhibition (Figure 2c,d).

### 3.7. Molecular Docking

**Figure 3:**
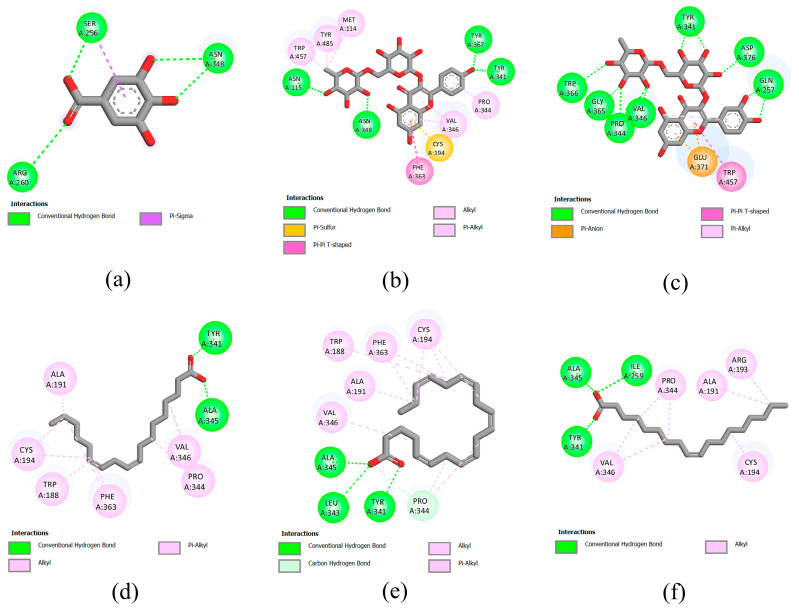
Molecular docking analysis of inducible iNOS (PDB ID: 3E6T). 2D diagrams of interaction between 3E6T and (**a**) gallic acid (positive control), (**b**) kaempferol-3-O-rutinoside, (**c**) rutin, (**d**) linoleic acid, (**e**) eicosapentaenoic acid, and (**f**) oleic acid.

**Figure 4:**
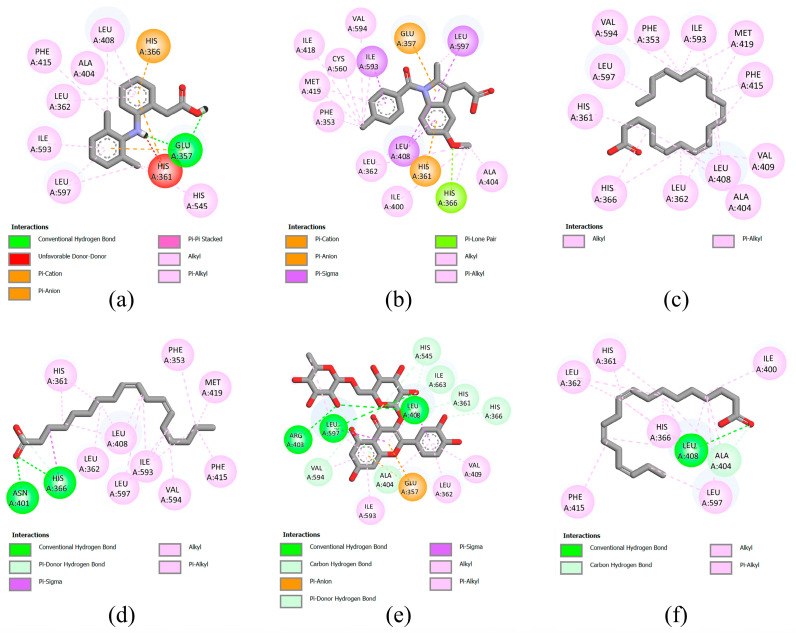
Molecular docking analysis of 15-LOX. 2D diagrams of interaction between 15-LOX and (**a**) sodium diclofenac (positive control), (**b**) indomethacin (positive control), (**c**) eicosapentaenoic acid, (**d**) linoleic acid, (**e**) rutin, (**f**) and linolenic acid positive control and compounds with the lowest binding energy.

**Figure 5:**
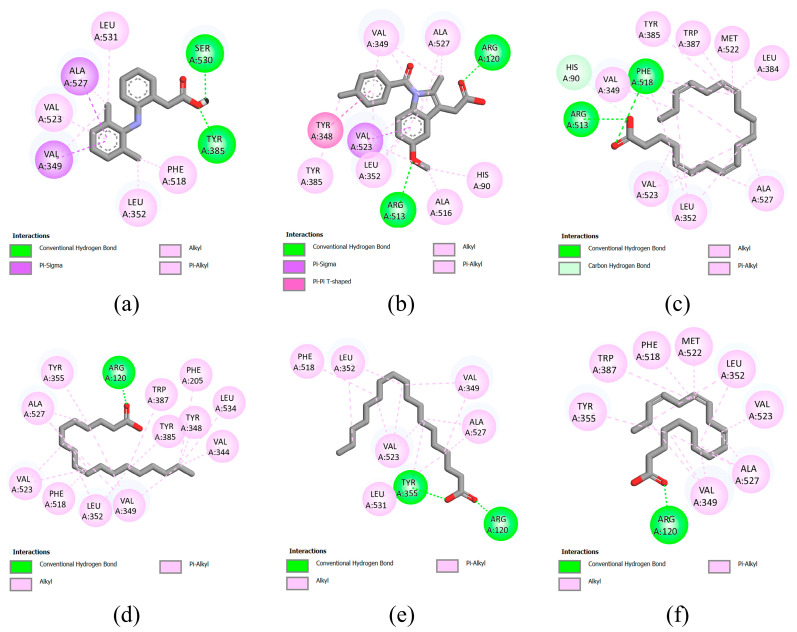
Molecular docking analysis of COX-2 (PDB ID: 5KIR). 2D diagrams of interaction between 5KIR and (**a**) sodium diclofenac (positive control), (**b**) indomethacin (positive control), (**c**) eicosapentaenoic acid, (**d**) oleic acid, (**e**) stearic acid, and (**f**) linolenic acid.

According to the results of previous in vitro experiments, the leaves of bananas exhibited the strongest anti-inflammatory activity, especially the ethyl acetate extract. Therefore, molecular docking was performed to investigate the potential interactions of phytochemicals in the leaves of bananas with the enzymes involved in the inflammatory response. Inducible iNOS, 15-LOX, and COX-2 enzymes were selected as targets for molecular docking. Information regarding the interactions between the target enzymes and compounds, including the binding energies, inhibition constants, and interacting amino acids, is presented in Supplementary Tables S3–S5. The two-dimensional (2D) diagrams of ligand–protein interactions for the positive controls and the five compounds with the lowest binding energies are shown in Figure 3, Figure 4 and Figure 5. The binding energy between the iNOS and compounds range from −3.09 to −9.77 kcal/mol (Supplementary Table S4). The compounds with binding energy lower than that of the positive control gallic acid (−3.96 kcal/mol) are kaempferol-3-*O*-rutinoside (−9.77 kcal/mol), rutin (−9.22 kcal/mol), linoleic acid (−4.97 kcal/mol), eicosapentaenoic acid (−4.93 kcal/mol), oleic acid (−4.44 kcal/mL), stearic acid (−4.33 kcal/mol), and palmitic acid (−4.27 kcal/mol). 

The binding energy between the 15-LOX and compounds range from −3.57–(−6.62) kcal/mol. Sodium diclofenac and indomethacin were used as positive controls, which show a binding energy of −6.87 and −7.73 kcal/mol, respectively (Supplementary Table S5). None of the compounds in banana leaves exhibited binding energies lower than those of the positive controls. Among the compounds in banana leaf, eicosapentaenoic acid shows the lowest binding energy (−6.62 kcal/mol), followed by linoleic acid (−5.94 kcal/mol), rutin (−5.91 kcal/mol), linolenic acid (−5.48 kcal/mol), and oleic acid (v5.17 kcal/mol).

Lastly, for the docking with COX-2 as a target, sodium diclofenac and indomethacin were used as positive controls, which show a binding energy of −7.42 and −8.68 kcal/mol, respectively (Supplementary Table S6). The binding energy between the COX-2 and compounds in banana leaf exhibit binding energies ranging from −3.81–(−7.26) kcal/mol, which is higher than that for the positive control. Among the compounds tested, eicosapentaenoic acid exhibit the lowest binding energy (−7.26 kcal/mol), followed by oleic acid (−6.87 kcal/mol), stearic acid (−6.69 kcal/mol), linolenic acid (−6.67 kcal/mol), and palmitic acid (−6.53 kcal/mol).

### 3.8. Brine Shrimp Lethality Assay

The toxicity of banana byproducts was assessed using a brine shrimp lethality assay. The results are presented in Supplementary Table S6. The water extract of ripe peel shows an LC_50_ of 469.39 µg/mL, followed by hexane extracts of peduncle and leaf with LC_50_ values of 737.89 and 802.39 µg/mL, respectively. However, the LC_50_ values for other extracts are higher than 1000 µg/mL, which are considered non-toxic. 

## 4. Discussion

Phytochemicals are compounds naturally occurring in plants as secondary metabolites and many of them are shown to possess a wide range of pharmacological activities. Previous studies have reported that the leaf and fruit peel of the banana plant (*Musa paradisiaca*) contains different classes of phytochemical compounds like phenolics, flavonoids, tannins, saponins, alkaloids, and fatty acids [3,25,36]. Consistently, the results of our study demonstrated that the extracts of five different parts of Nam Wah banana (*Musa paradisiaca* L.) byproducts also contain several previously reported phytochemical classes, including phenolic, flavonoid, tannin, saponin, and alkaloid. In addition, this study revealed the presence of steroids, diterpenes, triterpenes, and cardiac glycosides in all parts, whereas none was detected for anthraquinones. Among the tested extracts, the ethyl acetate extract of banana leaves was of particular interest because it contained the widest variety of phytochemicals. Interestingly, its total phenolic and flavonoid contents were the highest and triterpenes were found only in this extract. Compared to others, these compounds may be responsible for the profound activities shown by leaf ethyl acetate extract. However, further studies regarding the identification and isolation of bioactive constituents should be carried out using advanced quantitative analytical techniques such as liquid chromatography coupled to tandem mass spectrometry (LC–MS/MS) and high-performance liquid chromatography (HPLC).

In this present study, we also found that the ethyl acetate extract of Nam Wah banana leaves exhibited the strongest antioxidant capacity in free radical scavenging compared with other extracts, determined by the DPPH and FRAP assays. Various chronic illnesses, such as atherosclerosis, chronic obstructive pulmonary disease, Alzheimer’s disease, and cancer, have been linked to oxidative stress [37]. The development of oxidative stress is caused by the overproduction of free radicals or insufficient antioxidant capacity. Therefore, the endogenous generation and intake of exogenous antioxidants are important as they can scavenge free radicals, such as reactive oxygen species and reactive nitrogen species [38]. Phenolic compounds and flavonoids are crucial antioxidant phytochemicals due to their ability to donate hydrogen atoms or electrons to free radicals [35]. Consistent with this, our study showed that both total phenolic and flavonoid contents of banana byproducts were highly positively correlated with their antioxidant activities. Hence, the higher total phenolic and flavonoid content in the ethyl acetate extract of banana leaves explains why it possessed a stronger antioxidant effect than the other extracts. Future analysis using a kinetic approach on the rate constant and the stoichiometry of the scavenging reaction can help differentiate and understand their antioxidant behaviors/mechanisms [39]. Notably, the previous study on Indonesian banana (*M. paradisiaca*) leaves has reported that the highest antioxidant activity and total phenolic/flavonoid contents were obtained from the ethyl acetate extract, compared with hexane and ethanol extracts [40], highlighting the potential value of this byproduct extract for future development.

Inflammation is a biological reaction that occurs during cellular injury [41]. NO, produced by iNOS, plays an important role in the development and elevation of inflammatory responses [42]. In hypoxic signaling pathways, NO can also induce the generation of additional reactive nitrogen species, which may worsen the severity of inflammation [43]. Therefore, the inhibition of NO is a promising anti-inflammatory approach. Similar to the antioxidant study, our results demonstrated that the higher the total phenolic contents, the stronger the NO scavenging activity. However, compared with the total phenolic content, the correlation between the total flavonoid content and NO scavenging activity was weaker. Because phenolic compounds can be classified into flavonoid and non-flavonoid polyphenols [44], our results suggest that non-flavonoid phenolic compounds may be the major compounds responsible for the NO scavenging activity in banana byproducts. 15-LOX also mediates inflammation by generating leukotrienes [45]. Previous studies have reported that the disruption of 15-LOX can inhibit the production of proinflammatory cytokines, thereby reducing allergic inflammatory reactions in the airways [46]. In the present study, although the ethyl acetate extract of banana leaves exerted the highest inhibitory effect on 15-LOX, Pearson’s correlation analysis showed that the correlation between total phenolic/flavonoid content and 15-LOX inhibition was moderate. The inhibitory effect of banana byproducts on 15-LOX may not be solely mediated by phenolic compounds and flavonoids but may also be related to terpenoid compounds such as saponins, steroids, triterpenes, and diterpenes. This is not implausible because some terpenoid compounds (that is, bornyl acetate and limonene) have been reported to inhibit 15-LOX [47]. 

Docking analysis was performed in the present study to identify the phenolic compounds and flavonoids that are potential anti-inflammatory compounds in banana byproducts. In the docking analysis, the minimum binding energy was deemed as the preferred docking pose [48]. Our results showed that the minimum binding energies for some compounds, such as kaempferol-3-*O*-rutinoside and rutin, which are flavanol glycosides [49], were even lower than those for gallic acid (which served as a positive control). Therefore, it was predicted that these compounds may significantly interact with iNOS and may even inhibit iNOS activity. In this case, the level of NO can be decreased, leading to the alleviation of the inflammatory response [50]. In addition to iNOS, a docking analysis was performed using 15-LOX and COX-2 as binding targets. COX-2 is an inducible enzyme that generates prostaglandins, which are major inflammatory mediators in the body [41]. In agreement with the relatively weak correlation between total phenolic and flavonoid content and the anti-inflammatory effect, as mentioned previously, the docking results did not reveal any significant interactions between phenolic compounds and flavonoids on 15-LOX and COX-2. However, our findings suggested that eicosapentaenoic acid, an omega-3-polyunsaturated fatty acid present in banana leaves, interacts with both enzymes. Omega-3-polyunsaturated fatty acids are anti-inflammatory because they may serve as competitive substrates that inhibit the functions of COX and LOX [51]. Further studies are required to verify whether eicosapentaenoic acid is the active ingredient responsible for the anti-inflammatory effects of banana byproducts. 

Although banana byproducts have potential antioxidant and anti-inflammatory effects, they may not be useful unless they are safe. Therefore, in this study, a brine shrimp lethality assay was performed to verify its potential toxicity. Water extract of ripe peel and hexane extracts of peduncle and leaf showed LC_50_ values ranging from 469.39–802.39 µg/mL, but such LC_50_ values were typically considered low toxicity [52]. Except for these three extracts, all the other extracts of banana byproduct have LC_50_ over 1000 µg/mL, indicating that they were non-toxic. Our findings align with the few existing toxicological studies of *M. paradisiaca* byproducts. No apparent acute toxicity of leaf extracts from Indian bananas and peel extracts from Mexico bananas was observed in rodent models [53,54,55]. Nevertheless, an earlier study of Nigerian banana (*M. paradisiaca*) leaf has shown moderate acute toxicity of the aqueous fraction from ethanolic extract to mice [56].

## 5. Conclusions

In conclusion, we determined that different Nam Wah banana byproduct extracts have different antioxidant and anti-inflammatory capacities and toxicities. Among the 20 extracts tested, the ethyl acetate extract of banana leaves showed the most prominent antioxidant and anti-inflammatory activities. Most extracts did not exhibit toxicity. Phytochemicals responsible for the antioxidant and anti-inflammatory effects of banana byproducts include phenolic compounds and flavonoids. However, based on the results of the docking analysis, the contribution of other phytochemicals, such as eicosapentaenoic acid, should not be excluded. Overall, this study provides valuable data on Nam Wah banana byproducts, particularly the leaf and unripe peel, which were revealed as natural sources of powerful antioxidant and anti-inflammatory agents. Both parts should be further investigated for their therapeutic potential in oxidative stress- or inflammation-associated disorders. Banana byproducts should not be treated as waste because they are a potential source of antioxidants and anti-inflammatory compounds and thus have important health and economic value if fully utilized. 

## Figures and Tables

**Table 1:** Qualitative analysis of phytochemicals in banana byproducts.

Banana Byproducts	Extractions	Phenolic compounds	Flavonoids	Tannins	Diterpenes	Steroids	Triterpenes	Saponins	Alkaloids	Anthraquinones	Cardiac Glycosides
Midrib	Hexane	-	-	-	-	+	-	+	-	-	+
	Ethyl acetate	+	+	+	+	+	-	+	-	-	+
	Ethanol	+	+	-	+	+	-	+	-	-	-
	Water	+	+	-	+	+	-	+	-	-	-
Leaf	Hexane	-	-	-	-	+	-	-	-	-	+
	Ethyl acetate	+	+	+	+	+	+	+	+	-	+
	Ethanol	+	+	-	+	+	-	+	-	-	-
	Water	+	+	-	+	+	-	+	-	-	-
Peduncle	Hexane	-	-	-	-	+	-	-	-	-	+
	Ethyl acetate	+	+	-	+	+	-	+	+	-	+
	Ethanol	+	+	-	+	+	-	+	-	-	-
	Water	+	+	-	+	-	-	+	-	-	-
Unripe peel	Hexane	-	-	-	-	+	-	-	-	-	+
	Ethyl acetate	+	+	+	+	+	-	+	-	-	+
	Ethanol	+	+	-	+	+	-	+	-	-	-
	Water	+	-	-	-	+	-	+	-	-	-
Ripe peel	Hexane	-	-	-	-	+	-	-	-	-	+
	Ethyl acetate	+	+	-	+	+	-	+	+	-	+
	Ethanol	+	+	+	+	+	-	-	-	-	-
	Water	-	-	-	-	+	-	+	-	-	-

+ = present; - = absent.

**Table 2:** Total phenolic and flavonoid contents of banana byproducts.

Banana Byproducts	Extractions	Total Phenolic Contents (mg GAE/g Dry Extract)	Total Flavonoid Contents (mg QE/g Dry Extract)
Midrib	Hexane	4.70 ± 0.20 ^h^	2.55 ± 0.52 ^g,h^
	Ethyl acetate	20.56 ± 1.26 ^f^	11.18 ± 0.54 ^c,d^
	Ethanol	21.56 ± 0.75 ^f^	3.86 ± 0.33 ^g,h^
	Water	16.63 ± 0.81 ^f,g^	1.47 ± 0.23 ^h^
Leaf	Hexane	11.29 ± 0.77 ^g,h^	4.47 ± 0.47 ^f,g,h^
	Ethyl acetate	148.21 ± 12.04 ^a^	53.87 ± 5.38 ^a^
	Ethanol	49.85 ± 2.68 ^c^	14.15 ± 0.47 ^c^
	Water	47.73 ± 1.23 ^c^	22.16 ± 1.65 ^b^
Peduncle	Hexane	10.69 ± 0.36 ^g,h^	1.93 ± 0.34 ^h^
	Ethyl acetate	48.77 ± 1.59 ^c^	6.41 ± 0.62 ^e,f,g^
	Ethanol	31.61 ± 0.50 ^e^	1.63 ± 0.49 ^h^
	Water	16.85 ± 0.28 ^f,g^	1.03 ± 0.19 ^h^
Unripe peel	Hexane	3.11 ± 0.14 ^h^	n.d.
	Ethyl acetate	37.33 ± 1.11 ^d,e^	8.79 ± 0.80 ^d,e^
	Ethanol	59.14 ± 2.14 ^b^	2.07 ± 0.18 ^h^
	Water	2.71 ± 0.22 ^h^	1.05 ± 0.10 ^h^
Ripe peel	Hexane	2.78 ± 0.20 ^h^	2.59 ± 0.10 ^g,h^
	Ethyl acetate	45.17 ± 1.68 ^c,d^	8.04 ± 0.71 ^d,e,f^
	Ethanol	33.75 ± 1.76 ^e^	1.26 ± 0.06 ^h^
	Water	6.30 ± 0.60 ^h^	1.18 ± 0.08 ^h^

Values show mean ± SD of at least three independent experiments; GAE = gallic acid equivalent; QE = quercetin equivalent; n.d. = not detectable. Different letters in the same column indicate statistically significant differences (*p* < 0.05; ANOVA with Tukey’s post hoc test).

**Table 3:** DPPH radical scavenging activities and FRAP values of banana byproducts.

Banana Byproducts	Extractions	DPPH			FRAP
		Scavenging Activities (%)	IC_50_ (mg/mL)	mg VCEAC/g Dry Extract	mM AAE/mg Dry Extract
Midrib	Hexane		>5	2.62 ± 0.17 ^f,g^	1.77 ± 0.10 ^l,m^
	1 mg/mL	4.84 ± 0.36 ^k,l,m^			
	5 mg/mL	13.39 ± 1.26 ^g^			
	Ethyl acetate		4.546 ± 0.37 ^h^	26.84 ± 3.73 ^e^	7.60 ± 0.18 ^h,i^
	1 mg/mL	27.22 ± 2.56 ^e,f^			
	Ethanol		1.976 ± 0.06 ^d,e,f^	34.96 ± 2.44 ^d,e^	8.63 ± 0.37 ^h^
	1 mg/mL	34.57 ± 2.16 ^d^			
	Water		4.03 ± 0.11 ^g^	9.23 ± 0.33 ^f,g^	6.58 ± 0.40 ^i,j^
	1 mg/mL	13.03 ± 1.21 ^g,h^			
Leaf	Hexane		>5	3.57 ± 0.15 ^f,g^	5.21 ± 0.06 ^j,k^
	1 mg/mL	7.64 ± 1.01 ^i,j,k^			
	5 mg/mL	12.37 ± 0.99 ^g,h,i,j^			
	Ethyl acetate		0.251 ± 0.01 ^a^	235.10 ± 12.88 ^a^	70.74 ± 0.67 ^a^
	1 mg/mL	90.76 ± 2.76 ^a^			
	Ethanol		0.852 ± 0.06 ^b^	55.51 ± 5.11 ^b^	40.62 ± 1.11 ^b^
	1 mg/mL	56.01 ± 1.74 ^b^			
	Water		0.978 ± 0.03 ^b^	48.54 ± 7.98 ^b,c^	19.66 ± 0.30 ^e^
	1 mg/mL	53.05 ± 4.06 ^b^			
Peduncle	Hexane		>5	3.61 ± 0.28 ^f,g^	1.82 ± 0.07 ^l,m^
	1 mg/mL	4.58 ± 0.97 ^k,l,m^			
	5 mg/mL	13.51 ± 1.46 ^g^			
	Ethyl acetate		2.358 ± 0.17 ^f^	35.41 ± 2.04 ^d,e^	21.72 ± 1.56 ^d^
	1 mg/mL	27.28 ± 1.57 ^e,f^			
	Ethanol		1.944 ± 0.05 ^d,e^	27.37 ± 2.04 ^d,e^	12.53 ± 0.36 ^g^
	1 mg/mL	26.25 ± 1.92 ^e,f^			
	Water		>5	12.59 ± 0.18 ^f^	7.29 ± 0.20 ^h,i^
	1 mg/mL	12.40 ± 0.19 ^g,h,i^			
	5 mg/mL	41.41 ± 1.31 ^c^			
Unripe peel	Hexane		>5	2.29 ± 0.17 ^f,g^	3.35 ± 0.44 ^k,l^
	1 mg/mL	3.56 ± 1.07 ^k,l,m^			
	5 mg/mL	7.07 ± 0.38 ^j,k,l^			
	Ethyl acetate		1.786 ± 0.01 ^c,d^	33.91 ± 1.37 ^d,e^	17.21 ± 0.82 ^f^
	1 mg/mL	31.54 ± 2.02 ^d,e^			
	Ethanol		0.968 ± 0.06 ^b^	55.67 ± 2.27 ^b^	25.73 ± 1.21 ^c^
	1 mg/mL	52.85 ± 2.47 ^b^			
	Water		>5	1.00 ± 0.10 ^g^	1.26 ± 0.24 ^m^
	1 mg/mL	1.01 ± 0.13 ^m^			
	5 mg/mL	0.12 ± 0.14 ^m^			
Ripe peel	Hexane		>5	3.57 ± 0.19 ^f,g^	2.15 ± 0.19 ^l,m^
	1 mg/mL	3.80 ± 0.5 ^k,l,m^			
	5 mg/mL	12.45 ± 1.77 ^g,h,i^			
	Ethyl acetate		1.455 ± 0.10 ^c^	38.76 ± 2.87 ^c,d^	20.35 ± 0.66 ^d,e^
	1 mg/mL	36.29 ± 3.14 ^c,d^			
	Ethanol		2.277 ± 0.14 ^e,f^	26.12 ± 1.50 ^e^	13.55 ± 0.15 ^g^
	1 mg/mL	25.03 ± 1.89 ^f^			
	Water		>5	2.15 ± 0.24 ^f,g^	1.66 ± 0.08 ^l,m^
	1 mg/mL	2.15 ± 0.33 ^l,m^			
	5 mg/mL	8.04 ± 0.38 ^h,i,j,k^			

Values show mean ± SD of at least three independent experiments; IC_50_ is the concentration at which the 50% scavenging activity is observed; VCEAC = vitamin C equivalent antioxidant capacity; AAE = ascorbic acid equivalent. Different letters in the same column indicate statistically significant differences (*p* < 0.05; ANOVA with Tukey’s post hoc test).

**Table 4:** NO scavenging activities of banana byproducts.

Banana Byproducts	Extractions	Scavenging Activities (%)	IC_50_ (mg/mL)
Midrib	Hexane		>5
	1 mg/mL	n.d.	
	5 mg/mL	27.08 ± 2.30 ^e^	
	Ethyl acetate		>5
	1 mg/mL	2.93 ± 1.04 ^j^	
	5 mg/mL	42.01 ± 2.91 ^b,c^	
	Ethanol		>5
	1 mg/mL	n.d.	
	5 mg/mL	36.43 ± 0.09 ^b,c,d^	
	Water		>5
	1 mg/mL	n.d.	
	5 mg/mL	35.12 ± 4.17 ^c,d^	
Leaf	Hexane		2.744 ± 0.10 ^b^
	1 mg/mL	12.64 ± 1.16 ^f,g,h,i^	
	Ethyl acetate		0.782 ± 0.03 ^a^
	1 mg/mL	58.34 ± 2.23 ^a^	
	Ethanol		4.201 ± 0.05 ^c^
	1 mg/mL	11.98 ± 0.47 ^f,g,h,i^	
	Water		2.997 ± 0.08 ^b^
	1 mg/mL	16.85 ± 0.07 ^f,g^	
Peduncle	Hexane		>5
	1 mg/mL	n.d.	
	5 mg/mL	34.86 ± 3.15 ^c,d^	
	Ethyl acetate		>5
	1 mg/mL	8.94 ± 1.13 ^i,j^	
	5 mg/mL	42.84 ± 3.21 ^b^	
	Ethanol		4.202 ± 0.32 ^c^
	1 mg/mL	9.31 ± 1.23 ^h,i,j^	
	Water		>5
	1 mg/mL	n.d.	
	5 mg/mL	34.73 ± 3.11 ^d^	
Unripe peel	Hexane		>5
	1 mg/mL	n.d.	
	5 mg/mL	16.28 ± 1.58 ^f,g,h^	
	Ethyl acetate		2.859 ± 0.14 ^b^
	1 mg/mL	17.15 ± 0.73 ^f^	
	Ethanol		3.123 ± 0.18 ^b^
	1 mg/mL	11.97 ± 1.60 ^f,g,h,i^	
	Water		>5
	1 mg/mL	n.d.	
	5 mg/mL	n.d.	
Ripe peel	Hexane		>5
	1 mg/mL	n.d.	
	5 mg/mL	n.d.	
	Ethyl acetate		3.970 ± 0.35 ^c^
	1 mg/mL	9.66 ± 4.82 ^g,h,i,j^	
	Ethanol		4.380 ± 0.30 ^c^
	1 mg/mL	14.42 ± 2.53 ^f,g,h,i^	
	Water		>5
	1 mg/mL	n.d.	
	5 mg/mL	9.51 ± 1.02 ^h,i,j^	
Gallic acid			0.057 ± 0.003

Values show mean ± SD of at least three independent experiments; IC_50_ is the concentration at which the 50% scavenging activity is observed; n.d. = not detectable. Different letters in the same column indicate statistically significant differences (*p* < 0.05; ANOVA with Tukey’s post hoc test).

**Table 5:** 15-LOX inhibitory activities of banana byproducts.

Banana Byproducts	Extractions	% Inhibition (at 1 mg/mL)
Midrib	Hexane	10.68 ± 2.37 ^h,i^
	Ethyl acetate	34.23 ± 4.76 ^b,c,d^
	Ethanol	38.43 ± 1.52 ^b,c^
	Water	19.42 ± 3.97 ^f,g,h,i^
Leaf	Hexane	50.39 ± 5.72 ^a^
	Ethyl acetate	53.04 ± 2.20 ^a^
	Ethanol	35.07 ± 3.28 ^b,c,d^
	Water	15.11 ± 2.41 ^g,h,i^
Peduncle	Hexane	25.14 ± 9.29 ^d,e,f,g^
	Ethyl acetate	42.77 ± 8.63 ^a,b^
	Ethanol	21.82 ± 3.17 ^e,f,g,h,i^
	Water	21.18 ± 1.66 ^e,f,g,h,i^
Unripe peel	Hexane	20.29 ± 3.54 ^e,f,g,h,i^
	Ethyl acetate	20.42 ± 3.43 ^e,f,g,h,i^
	Ethanol	21.23 ± 2.52 ^e,f,g,h,i^
	Water	10.13 ± 0.89 ^i^
Ripe peel	Hexane	26.71 ± 2.80 ^d,e,f,g^
	Ethyl acetate	30.06 ± 2.56 ^c,d,e,f^
	Ethanol	31.50 ± 2.66 ^b,c,d,e^
	Water	22.15 ± 2.77 ^e,f,g,h^
Sodium diclofenac		42.18 ± 1.47

Values show mean ± SD of at least three independent experiments. Different letters in the same column indicate statistically significant differences (*p* < 0.05; ANOVA with Tukey’s post hoc test).

## Data Availability

The data generated or analyzed during the current study that are relevant to the results presented here have been included in this article and its supplementary information file.

## Supplementary Materials

The following supporting information can be downloaded at: https://www.mdpi.com/article/10.3390/foods12213955/s1, Table S1: List of phytochemical compounds reported in leaves of Musa spp.; Table S2: Extraction yields of banana byproducts; Table S3: Molecular docking results between compounds from *Musa* spp. leaves and the binding site of inducible nitric oxide synthases (iNOS) (PDB ID: 3E6T); Table S4: Molecular docking results between compounds from *Musa* spp. leaves and the binding site of 15-lipoxygenase (15-LOX) (PDB ID: 1LOX); Table S5: Molecular docking results between compounds from *Musa* spp. leaves and the binding site of cyclooxygenase-2 (COX-2) (PDB ID: 5KIR); Table S6: Toxicity of banana byproducts in brine shrimp lethality test.
