# Supplementary material for: Valorization of Nam Wah Banana (Musa paradisiaca L.) Byproducts as a Source of Bioactive Compounds with Antioxidant and Anti-inflammatory Properties: In Vitro and In Silico Studies
Source: Foods. 2023 Oct 29;12(21):3955. doi: 10.3390/foods12213955 (PMC10649638; doi:10.3390/foods12213955)
Supplement: Supplementary file 1 [file foods-12-03955-s001.zip › foods-2666635-supplementary.pdf]

Supplementary Materials

Valorization of Nam Wah Banana (*Musa paradisiaca* L.) Byproducts as a Source of Bioactive Compounds with Antioxidant and Anti-inflammatory Properties: In Vitro and In Silico Studies

Supplementary Table S1. List of phytochemical compounds reported in leaves of Musa spp.

Compound	Source	Structure	Reference
Behenic acid	*M. acuminata* X *M. balbisiana* (ABB group)	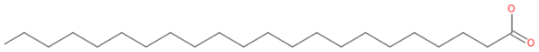	[1]
Eicosapentaenoic acid	*M. acuminata* X *M. balbisiana* (ABB group)	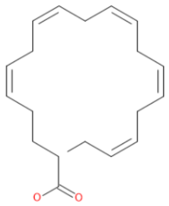	[1]
Kaempferol-3-O-rutinoside	*M. balbisiana*	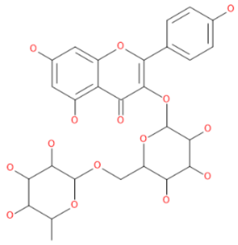	[2]
Lignoceric acid	*M. acuminata* X *M. balbisiana* (ABB group)	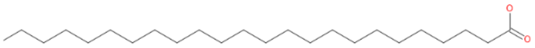	[1]
Linoleic acid	*M. acuminata* X *M. balbisiana* (ABB group), *Musa liukiuensis, M. acuminate,* and *M. chiliocarpa*	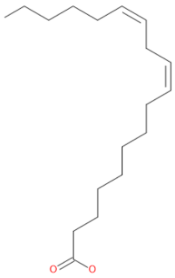	[1, 3]

Linolenic acid	*M. acuminata* X *M. balbisiana* (ABB group)	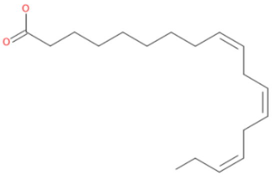	[1]
Oleic acid	*M. acuminata* X *M. balbisiana* (ABB group)	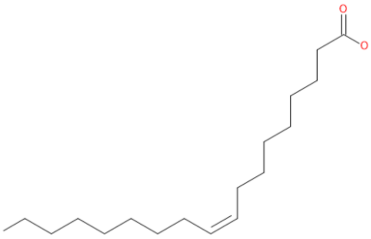	[1]
Palmitic acid	*M. acuminata* X *M. balbisiana* (ABB group), *Musa liukiuensis, M. acuminate,* and *M. chiliocarpa*	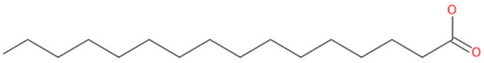	[1, 3]
Rutin	*M. balbisiana* and *M. paradisiaca*	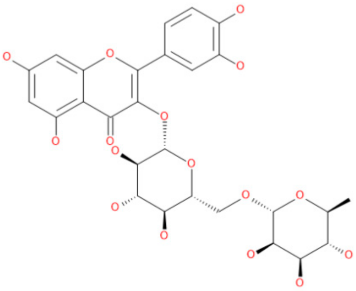	[2, 4]
Stearic acid	*M. acuminata* X *M. balbisiana* (ABB group), *Musa liukiuensis, M. acuminate,* and *M. chiliocarpa*	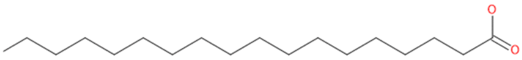	[1, 3]

Supplementary Table S2. Extraction yields of banana byproducts.

Banana byproducts	Extraction method / Solvent	Yield (%)
Midrib	Soxhlet / Hexane	4.22
	Soxhlet / Ethyl Acetate	1.30
	Soxhlet / Ethanol	19.31
	Maceration / Water	18.18
Leaf	Soxhlet / Hexane	9.00
	Soxhlet / Ethyl Acetate	7.46
	Soxhlet / Ethanol	17.90
	Maceration / Water	10.44
Peduncle	Soxhlet / Hexane	1.37
	Soxhlet / Ethyl Acetate	2.75
	Soxhlet / Ethanol	15.29
	Maceration / Water	24.34
Unripe peel	Soxhlet / Hexane	11.32
	Soxhlet / Ethyl Acetate	1.27
	Soxhlet / Ethanol	11.13
	Maceration / Water	13.47
Ripe peel	Soxhlet / Hexane	12.61
	Soxhlet / Ethyl Acetate	1.38
	Soxhlet / Ethanol	22.35
	Maceration / Water	23.46

Supplementary Table S3. Molecular docking results between compounds from Musa spp. leaves and the binding site of inducible nitric oxide synthases (iNOS) (PDB ID: 3E6T).

No.	Compound	Binding energy (kcal/mol)	Inhibition Constant	Amino acid interaction		
				Hydrogen bond	Hydrophobic bond	Electrostatic bond
	5-(4'-Amino-1'-ethyl-5',8'-difluoro-1'H-spiro [piperidine-4,2'-quinazoline]-1-ylcarbonyl) picolinonitrile (Original ligand re-docking)	-7.39	3.85 µM	TYR341<br>GLY365<br>TRP366<br>GLU371<br>GLU371<br>ASP376	PRO344<br>PRO344<br>TYR367	ARG260
	Gallic acid (Positive control)	-3.96	1.25 mM	SER256<br>ARG260<br>ASN348<br>ASN348	SER256	
1	Kaempferol-3-O-rutinoside	-9.77	69.38 nM	ASN115<br>ASN115<br>TYR341<br>ASN348<br>TYR367	MET114<br>PRO344<br>VAL346<br>VAL346<br>PHE363<br>TRP457<br>TYR485	CYS194
2	Rutin	-9.22	174.97 nM	GLN257<br>GLN257<br>TYR341<br>TYR341<br>PRO344<br>PRO344<br>VAL346<br>GLY365<br>TRP366<br>ASP376	GLU371<br>GLU371	VAL346<br>TRP457
3	Linoleic acid	-4.97	228.11 µM	TYR341<br>ALA345	TRP188<br>TRP188<br>ALA191<br>CYS194<br>CYS194<br>PRO344<br>PRO344<br>VAL346	

					PHE363
					PHE363
4	Eicosapentaenoic acid	-4.93	242.01 µM	TYR341	TRP188
				LEU343	ALA191
				PRO344	CYS194
				ALA345	CYS194
					CYS194
					PRO344
					PRO344
					VAL346
					PHE363
					PHE363
5	Oleic acid	-4.44	553.02 µM	ILE259	ALA191
				TYR341	ARG193
				ALA345	CYS194
					PRO344
					PRO344
					VAL346
					VAL346
6	Linolenic acid	-4.36	632.09 µM	TYR341	CYS194
				ALA345	CYS194
					PRO344
					PRO344
					PRO344
					VAL346
7	Stearic acid	-4.33	670.63 µM	TYR341	TRP188
				ALA345	ARG193
					CYS194
					PRO344
					PRO344
					VAL346
					PHE363
					PHE363
8	Palmitic acid	-4.27	737.18 µM	TYR341	ALA191
				ALA345	CYS194
				LEU343	PRO344
					PRO344
					VAL346
					VAL346
					PHE363
					PHE363

					TYR367
9	Behenic acid	-3.92	1.33 mM		CYS194
					CYS194
					PRO344
					PRO344
					PRO344
					VAL346
					VAL346
					VAL346
					PHE363
					PHE363
					TYR367
10	Lignoceric acid	-3.09	5.44 mM	TYR341	ALA191
					ARG193
					CYS194
					CYS194
					PRO344
					PRO344
					PRO344
					VAL346
					TYR367
					TRP457

Supplementary Table S4. Molecular docking results between compounds from Musa spp. leaves and the binding site of 15-lipoxygenase (15-LOX) (PDB ID: 1LOX).

**No.**	**Compound**	**Binding energy (kcal/mol)**	**Inhibition Constant**	**Amino acid interaction**		
				**Hydrogen bond**	**Hydrophobic bond**	**Electrostatic bond**
	(2e)-3-(2-Oct-1-Yn-1-Ylphenyl)acrylic Acid (Original ligand re-docking)	-6.36	21.64 µM		HIS361<br>LEU362<br>HIS366<br>HIS366<br>LEU408<br>ALA404<br>ILE593<br>LEU597	GLU357
	Sodium Diclofenac (Positive control)	-6.87	9.17 µM	GLU357<br>GLU357	HIS361<br>LEU362<br>HIS366<br>ALA404<br>LEU408<br>LEU408<br>PHE415<br>HIS545<br>ILE593<br>LEU597<br>LEU597	GLU357<br>HIS361<br>HIS366
	Indomethacin (Positive control)	-7.73	2.14 µM		PHE353<br>LEU362<br>LEU362<br>HIS366<br>ILE400<br>ALA404<br>LEU408<br>LEU408<br>ILE418<br>MET419<br>MET419<br>CYS560<br>ILE593<br>ILE593<br>ILE593<br>VAL594	GLU357<br>HIS361

					LEU597	
					LEU597	
1	Eicosapentaenoic acid	-6.62	25.63 µM		HIS361	
					PHE353	
					LEU362	
					LEU362	
					HIS366	
					HIS366	
					ALA404	
					LEU408	
					LEU408	
					LEU408	
					LEU408	
					VAL409	
					PHE415	
					PHE415	
					MET419	
					MET419	
					ILE593	
					ILE593	
					VAL594	
					LEU597	
2	Linoleic acid	-5.94	43.94 µM	HIS366	PHE353	
				HIS366	HIS361	
				ASN401	HIS361	
					LEU362	
					HIS366	
					LEU408	
					LEU408	
					LEU408	
					PHE415	
					MET419	
					ILE593	
					ILE593	
					ILE593	
					VAL594	
					LEU597	
					LEU597	
3	Rutin	-5.91	46.71 µM	HIS361	ALA404	GLU357
				HIS366	LEU408	GLU357
				ARG403	LEU408	

				ALA404	VAL409
				LEU408	ILE593
				HIS545	VAL594
				HIS545	LEU597
				VAL594	LEU597
				LEU597	LEU597
				LEU597	LEU362
				ILE663	
4	Linolenic acid	-5.48	95.93 µM	ALA404	HIS361
				LEU408	HIS361
					LEU362
					LEU362
					HIS366
					HIS366
					ILE400
					ALA404
					ALA404
					LEU408
					LEU408
					LEU408
					LEU408
					PHE415
					LEU597
					LEU597
5	Oleic acid	-5.17	161.95 µM		PHE353
					HIS361
					HIS361
					HIS361
					LEU362
					LEU362
					HIS366
					HIS366
					ALA404
					LEU408
					LEU408
					LEU408
					MET419
					ILE593
					ILE593
					VAL594
					LEU597

6	Stearic acid	-4.71	353.03 µM	HIS361<br>LEU362<br>LEU362<br>HIS366<br>HIS366<br>ILE400<br>ARG403<br>ALA404<br>ALA404<br>ALA404<br>VAL409<br>LEU408<br>LEU408<br>LEU408<br>LEU408<br>PHE415<br>LEU597
7	Palmitic acid	-4.57	443.12 µM	HIS361<br>HIS361<br>LEU362<br>LEU362<br>HIS366<br>HIS366<br>ILE400<br>ALA404<br>LEU408<br>LEU408<br>LEU408<br>PHE415<br>ILE593<br>LEU597
8	Behenic acid	-4.42	579.68 µM	PHE353<br>HIS361<br>HIS361<br>LEU362<br>LEU362<br>HIS366<br>HIS366<br>ILE400<br>ALA404<br>ALA404

					LEU408	
					LEU408	
					LEU408	
					ILE418	
					MET419	
					ILE593	
					ILE593	
					ILE593	
					LEU597	
					LEU597	
					LEU597	
9	Lignoceric acid	-4.29	711.91 µM		PHE353	
					HIS361	
					HIS361	
					LEU362	
					LEU362	
					HIS366	
					HIS366	
					ILE400	
					ILE400	
					ARG403	
					ALA404	
					ALA404	
					LEU408	
					LEU408	
					LEU408	
					LEU408	
					LEU408	
					PHE415	
					ILE418	
					MET419	
					ILE593	
					ILE593	
					ILE593	
					VAL594	
					LEU597	
					LEU597	
10	Kaempferol-3-O-rutinoside	-3.57	2.42 mM	HIS361	GLU357	GLU357
				HIS366	LEU358	GLU357
				ILE400	LEU358	
				ASN401	LEU362	

	HIS545	ALA404
	GLN548	LEU408
	LEU597	LEU408
		LEU408
		VAL409
		ILE593
		VAL594
		LEU597
		LEU597

Supplementary Table S5. Molecular docking results between compounds from *Musa* spp. leaves and the binding site of cyclooxygenase-2 (COX-2) (PDB ID: 5KIR).

No.	Compound	Binding energy (kcal/mol)	Inhibition Constant	Amino acid interaction		
				Hydrogen bond	Hydrophobic bond	Electrostatic bond
	Rofecoxib	-10.65	15.62 nM	ARG513	LEU352	
	(Original ligand re-docking)			ILE517	LEU352	
				PHE518	VAL349	
				ALA527	PHE518	
					VAL523	
					VAL523	
					VAL523	
					GLY526	
					ALA527	
					ALA527	
	Sodium Diclofenac	-7.42	3.65 µM	TYR385	VAL349	
	(Positive control)			SER530	VAL349	
					LEU352	
					PHE518	
					VAL523	
					ALA527	
					ALA527	
					LEU531	
	Indomethacin	-8.68	433.96 nM	ARG120	HIS90	
	(Positive control)			ARG120	TYR348	
				ARG513	VAL349	
					VAL349	
					VAL349	
					LEU352	
					LEU352	
					LEU352	
					TYR385	
					ALA516	
					VAL523	
					VAL523	
					VAL523	
					VAL523	
					ALA527	
					ALA527	
1	Eicosapentaenoic acid	-7.26	4.76 µM	HIS90	VAL349	

				ARG513	LEU352
				ARG513	LEU352
				PHE518	LEU352
					LEU384
					TYR385
					TRP387
					TRP387
					PHE518
					PHE518
					PHE518
					MET522
					VAL523
					VAL523
					VAL523
					ALA527
					ALA527
2	Oleic acid	-6.87	9.28 µM	ARG120	PHE205
				ARG120	VAL344
					TYR348
					VAL349
					VAL349
					VAL349
					LEU352
					LEU352
					TYR355
					TYR385
					TRP387
					PHE518
					VAL523
					VAL523
					ALA527
					ALA527
					ALA527
					LEU534
3	Stearic acid	-6.69	12.44 µM	ARG120	VAL349
				TYR355	VAL349
					LEU352
					LEU352
					TYR355
					PHE518
					VAL523

					VAL523
					VAL523
					ALA527
					ALA527
					LEU531
4	Linolenic acid	-6.67	12.89 µM	ARG120	VAL349
				ARG120	VAL349
					VAL349
					LEU352
					LEU352
					TYR355
					TRP387
					PHE518
					MET522
					VAL523
					ALA527
					ALA527
					ALA527
5	Palmitic acid	-6.53	16.38 µM	HIS90	VAL344
				TYR355	TYR348
				ARG513	VAL349
				ARG513	LEU352
					LEU352
					TRP387
					PHE518
					PHE518
					VAL523
					VAL523
					ALA527
6	Linoleic acid	-6.40	20.19 µM	ARG120	VAL349
				ARG120	LEU352
					LEU352
					LEU352
					TYR355
					LEU384
					TYR385
					TRP387
					TRP387
					PHE518
					MET522
					VAL523

					VAL523
					ALA527
7	Behenic acid	-5.79	56.72 µM	HIS90	VAL116
				HIS90	ARG120
				HIS90	VAL349
				ARG513	VAL349
				ARG513	LEU352
					LEU352
					LEU352
					TYR355
					PHE518
					PHE518
					VAL523
					VAL523
					VAL523
					VAL523
					ALA527
					ALA527
					ALA527
					ALA527
					LEU531
8	Kaempferol-3-O-rutinoside	-5.55	85.77 µM	HIS90	VAL116
				LEU352	VAL349
				SER353	VAL349
				TYR385	VAL349
				PHE518	TYR355
				MET522	LEU359
				GLY526	TYR385
					TRP387
					LEU531
					ALA527
					ALA527
					ALA527
					LEU531
9	Rutin	-4.71	351.45 nM	HIS90	VAL349
				ARG120	LEU352
				ARG120	LEU352
				GLN192	ALA516
				LEU352	ILE517
				TYR355	PHE518
				TYR355	VAL523

				ARG513	VAL523
				ARG513	ALA527
				ARG513	LEU531
				ILE517	
				PHE518	
				SER530	
10	Lignoceric acid	-3.81	1.60 mM		HIS90
					TYR348
					VAL349
					VAL349
					VAL349
					LEU352
					LEU352
					TYR385
					TRP387
					PHE518
					VAL523
					VAL523
					VAL523
					VAL523
					ALA527
					ALA527
					ALA527

Supplementary Table S6. Toxicity of banana byproducts in brine shrimp lethality test.

Banana byproducts	Solvent	Percentage of death at 24 hours					LC_{50} (µg/mL)
		10 (µg/mL)	100 (µg/mL)	250 (µg/mL)	500 (µg/mL)	1000 (µg/mL)	
Midrib	Hexane	0	0	0	0	12	> 1000
	Ethyl Acetate	0	0	0	0	10	> 1000
	Ethanol	0	0	2	4	6	> 1000
	Water	0	0	0	0	4	> 1000
Leaf	Hexane	0	0	2	10	86	802.39
	Ethyl Acetate	0	0	0	0	2	> 1000
	Ethanol	0	0	0	2	18	> 1000
	Water	0	0	0	6	26	> 1000
Peduncle	Hexane	0	0	8	24	84	737.89
	Ethyl Acetate	0	0	0	0	6	> 1000
	Ethanol	0	0	0	6	16	> 1000
	Water	0	0	12	14	34	> 1000
Unripe peel	Hexane	0	0	0	6	38	> 1000
	Ethyl Acetate	0	0	0	0	0	> 1000
	Ethanol	0	0	0	2	6	> 1000
	Water	0	0	4	14	36	> 1000
Ripe peel	Hexane	0	0	0	0	18	> 1000
	Ethyl Acetate	0	0	0	0	2	> 1000
	Ethanol	0	0	0	0	0	> 1000
	Water	0	0	6	56	94	469.39

References

1. Charumanee S, Yotsawimonwat S, Sirisa-Ard P, Pholsongkram K. Characterization and chemical composition of epicuticular wax from banana leaves grown in Northern Thailand. Songklanakarin Journal of Science & Technology. 2017;39(4).

2. Yingyuen P, Sukrong S, Phisalaphong M. Isolation, separation and purification of rutin from Banana leaves (Musa balbisiana). Industrial Crops and Products. 2020;149:112307.

3. Asemave K, Ujah SA. Determination of the Chemical Composition of Banana Leaves Wax. International Journal of Applied Sciences: Current and Future Research Trends. 2021;11(1):23-30.

4. Kappel VD, Cazarolli LH, Pereira DF, Postal BG, Madoglio FA, Buss ZdS, et al. Beneficial effects of banana leaves (Musa x paradisiaca) on glucose homeostasis: Multiple sites of action. Revista Brasileira de Farmacognosia. 2013;23(4):706-15.
